# Arthropod‐Related Health Threats (ARHTs) and Nuisance Arthropods in North Khorasan Province, Iran: A Comprehensive Review

**DOI:** 10.1155/jotm/8859034

**Published:** 2026-10-21

**Authors:** Hasan Bakhshi, Kourosh Arzamani, Hamidreza Shoraka, Fatemeh Kouhestani, Aboozar Soltani, Jalil Nejati, Alireza Omidi Oskouei, Abedin Saghafipour

**Affiliations:** ^1^ Arthropod Borne Diseases Research Center Ardabil University of Medical Sciences Ardabil Iran arums.ac.ir; ^2^ Vector-Borne Diseases Research Center North Khorasan University of Medical Sciences Bojnurd Iran nkums.ac.ir; ^3^ Department of Medical Entomology and Vector Control School of Health Research Center for Health Sciences Institute of Health Shiraz University of Medical Sciences Shiraz Iran sums.ac.ir; ^4^ Health Promotion Research Center Zahedan University of Medical Sciences Zahedan Iran zaums.ac.ir; ^5^ Department of Public Health Faculty of Health Qom University of Medical Sciences Qom Iran muq.ac.ir

**Keywords:** arthropod, health, Iran, threats, vector

## Abstract

The arthropod vectors, such as mosquitoes, sand flies, and ticks, transmit various pathogens to humans and animals, leading to public health issues. Therefore, an effective disease surveillance system is essential to provide information on these diseases in order to monitor their trends worldwide. The current study focused on the prevalence of arthropod‐borne diseases and their vectors in North Khorasan Province located in northeastern Iran, bordering Turkmenistan. A narrative literature review was conducted to analyze the status of arthropod‐related health threats (ARHTs) and their vectors in this area. Sources with no time limit up to 2025 were examined from PubMed, Google Scholar, ScienceDirect, and Web of Science databases using keywords like vector, arthropod, North Khorasan, and Iran. After excluding duplicates and irrelevant content, a final list of 31 papers was thoroughly evaluated. This province exhibits the occurrence of several major ARHTs, including visceral leishmaniasis, cutaneous leishmaniasis, West Nile fever, chikungunya, Crimean–Congo hemorrhagic fever, Q fever, *Theileria* infection, equine piroplasmosis, dirofilariasis, *Dipylidium caninum* infection, fascioliasis, hymenolepiasis, scorpionism, spider bites, pediculosis, and myiasis. Given the complex and diverse ecosystem of North Khorasan, implementing tailored and innovative strategies is essential for effective surveillance and early detection of arthropod‐borne diseases, thus enabling timely and efficient management of these health threats.

## 1. Introduction

The arthropod‐related health threats (ARHTs) are caused by various pathogens, including bacteria, viruses, protozoa, and helminths, which are transmitted by arthropods such as mosquitoes, ticks, and fleas. For instance, malaria, leishmaniasis, river blindness, sleeping sickness, plague, Lyme disease, Crimean–Congo hemorrhagic fever (CCHF), dengue fever, and Zika are parasitic, bacterial, and viral human diseases. These diseases are common arthropod‐borne diseases and critical public health concerns in endemic regions of the world, causing more than 700,000 deaths each year [1, 2]. According to the World Health Organization reports, about one in six individuals may be affected by these diseases at any given time worldwide [3]. For instance, malaria causes approximately 249 million cases globally and over 600,000 deaths annually [4].

Globally, the ARHTs represent a major global health challenge, and Iran’s diverse ecosystems and cross‐border interactions make it particularly vulnerable to these emerging threats [4]. In Iran, as a Middle Eastern country with diverse ecosystems, several vector‐borne diseases have been documented, including malaria [5], leishmaniasis [6], CCHF [7], chikungunya [8], and West Nile fever [9]. Additionally, ARHTs such as scorpionism [10], myiasis [11], pediculosis [12], scabies [13], loxoscelism [14], and envenomation by other spider species [15] have been reported. The incidence and mortality rates of arthropod‐related diseases vary in different regions of Iran. Therefore, for example, pediculosis is prevalent in all 31 provinces of Iran. Its trend was evaluated from 1.7% to 3.42% among urban students during 2014 to 2018 [16]. Furthermore, scorpions cause approximately 45,000 to 50,000 cases of scorpionism annually in Iran [17].

Given this wide range of diseases and their high incidence, comprehensive surveillance of vector‐borne diseases in high‐risk provinces is essential for effective control. One of these provinces that has a great diversity in vector‐borne diseases and their vectors is North Khorasan. In addition, it is one of the transit sites of domestic animals from the eastern borders (Afghanistan and Pakistan) into the Country. Given that many people in this province are engaged in livestock farming and are in contact with insects and arthropods, and that nomads migrate to this province from neighboring provinces every year, the residents of this area will be exposed to ectoparasites and diseases related to them. With this in mind, the present review identifies the spatial distribution of ARHTs and their vectors in North Khorasan Province, a northeastern Iranian region bordering Turkmenistan.

## 2. Materials and Methods

### 2.1. Study Area

#### 2.1.1. Geographical and Climatic Context of North Khorasan Province

North Khorasan Province spans approximately 28,434 km^2^ with a population of around 863,000 inhabitants. The provincial capital, Bojnurd, along with other counties such as Shirvan, Esfarayen, and Jajarm, exhibits a semiarid climate with distinct continental influences. The region’s climatic patterns are significantly shaped by variations in altitude and topography, creating diverse ecological niches that influence the distribution and activity of disease vectors (Figure 1).

**FIGURE 1:**
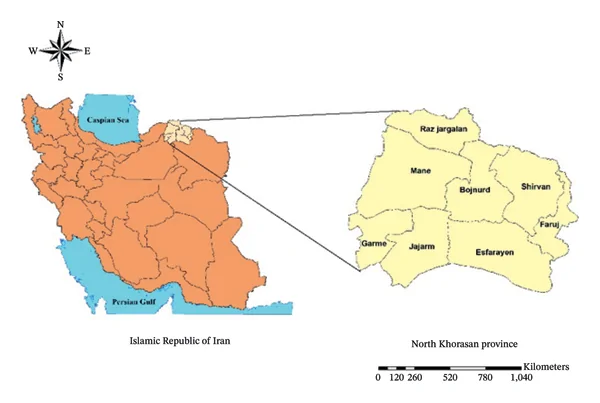
Map of study area: North Khorasan Province in northeastern Iran.

#### 2.1.2. Climate and Ecological Drivers of Vector‐Borne Diseases

Long‐term meteorological observations, as documented by Soltani et al., highlight the province’s semiarid characteristics [18]. Annual precipitation averages between 250 and 300 mm, with the majority of rainfall occurring during winter and spring months. This relatively low and seasonal precipitation pattern is typical of semiarid regions, where water availability becomes a critical factor in shaping vector breeding habitats. The limited rainfall results in intermittent water sources, which can concentrate vector populations around permanent or artificial water reservoirs, thereby influencing disease transmission dynamics.

Temperature patterns across the province further underscore its climatic diversity. Mean annual temperatures range from 13.2°C in Bojnurd, situated at 1091 m above sea level, to 16.4°C in lower elevation areas. Notably, minimum temperatures in Bojnurd have shown a significant increasing trend of 0.29°C per decade, a phenomenon attributed to both urbanization and broader greenhouse effects. Such warming trends have the potential to prolong the activity periods of arthropod vectors, thereby extending the transmission windows for vector‐borne diseases.

Humidity and evaporation rates also play a pivotal role in shaping the region’s vector ecology. Relative humidity averages around 55% in Bojnurd but can drop 40% below in southern districts, creating arid conditions that may limit vector survival outside of favorable microhabitats. The province experiences high potential evapotranspiration, peaking at 134 mm per month in July, which exacerbates water scarcity during the warmer months. These conditions restrict vector breeding to localized water sources, often in proximity to human settlements, where artificial water storage provides stable habitats for vectors such as mosquitoes [18–20].

#### 2.1.3. Topographic and Anthropogenic Influences

The varied topography of the province, with elevations ranging from 999 to 1491 m, generates microclimates that support distinct vector communities. For instance, cooler temperatures at higher elevations may favor tick populations, while warmer lowland areas provide suitable conditions for mosquitoes, particularly in areas with artificial water storage. Additionally, prolonged droughts in neighboring regions, such as Sistan and Baluchestan, have introduced complex climatic interactions, including aerosol‐driven cooling effects observed in Birjand, where maximum temperatures have decreased by 0.26°C per decade. These regional climatic anomalies further complicate the ecological dynamics of vector populations, as well as their associated disease risks [18–20].

#### 2.1.4. Implications for Disease Dynamics

The interplay of semiarid conditions, erratic rainfall, and rising temperatures in North Khorasan creates a mosaic of vector habitats that are often fragmented and closely tied to human activity. Seasonal water scarcity tends to concentrate vector breeding sites near populated areas, leading to an increase in the risk of disease transmission. Understanding these climatic and ecological drivers is indispensable for designing effective vector surveillance and control programs, as well as for predicting potential shifts in disease patterns under ongoing climate change [18–20].

### 2.2. Search Strategy

A narrative literature review was conducted to assess the status and public health importance of vector‐borne diseases in North Khorasan Province. Four major databases, including PubMed, Google Scholar, ScienceDirect, Web of Science, SID, and Magiran, were thoroughly searched using relevant keywords, such as “vector,” “arthropod,” “North Khorasan,” and “Iran,” with no time restrictions up to the year 2025. After removing duplicates and irrelevant records, a team of local and academic experts conducted a thorough evaluation of the final list of 31 eligible studies (Figure 2).

**FIGURE 2:**
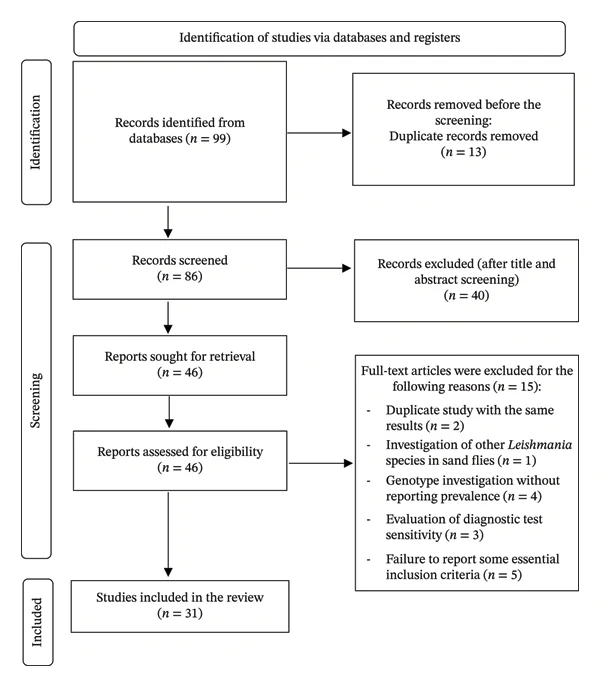
The flowchart of the literature review on ARHTs in North Khorasan Province, Iran.

#### 2.2.1. Article Selection Criteria

To ensure a systematic and unbiased review process, the following inclusion and exclusion criteria were employed.

Inclusion criteria were as follows:

• Studies published in peer‐reviewed journals reporting original research or comprehensive reviews related to arthropod‐borne diseases and associated complications in North Khorasan Province.
• Articles published in English or Persian up to 2025.
• Studies presenting clear methodologies and sufficient data relevant to vector‐borne diseases.

### 2.3. Data Extraction and Quality Assessment

Data were extracted systematically using a predefined extraction sheet, capturing details such as study location, disease type, incidence, primary vectors, reservoirs, and key findings. Study quality was assessed using the Newcastle–Ottawa Scale (NOS) for observational studies, with a threshold score of ≥ 6/9 for inclusion. NOS evaluations were conducted independently by two authors. In addition to the NOS, a supplementary quality assessment checklist was applied (Table 1). As not all criteria were equally applicable to descriptive epidemiological studies (e.g., sensitivity analysis), such items were recorded as NA (not applicable) or NR (not reported), as appropriate.

**TABLE 1:** Quality assessment of the 31 included studies: detailed criteria and score.

First author, year (ref.no^∗^)	Q1	Q2	Q3	Q4	Q5	Q6	Q7	Q8	Q9	Q10	Q11	Quality (score)
Bakhshi, 2020 [8]	✓	✓	✓	✓	✓	✓	✓	✓	✓	✓	✓	Good
Bakhshi, 2021 [9]	✓	✓	✓	✓	✓	✓	✓	✓	✓	✓	✓	Good
Mahmoudzadeh‐Niknam, 2012 [21]	✓	✓	✓	✓	✓	NR	✓	✓	✓	NR	NR	Fair
Rajabzadeh, 2015 [22]	✓	✓	✓	✓	✓	NR	NR	✓	✓	NR	NR	Fair
Mohammadiha, 2018 [23]	✓	✓	✓	✓	✓	✓	✓	✓	NR	✓	✓	Good
Shafiei, 2021 [24]	✓	✓	✓	✓	✓	✓	✓	✓	NR	✓	✓	Good
Lakzaei, 2025 [25]	✓	✓	✓	✓	✓	✓	NR	✓	NR	✓	✓	Good
Hashemi, 2018 [26]	✓	NR	✓	✓	✓	NR	✓	✓	✓	NR	✓	Good
Badirzadeh, 2018 [27]	✓	NR	✓	✓	✓	NR	NR	✓	NR	✓	✓	Fair
Javadian, 1976 [28]	✓	NR	✓	✓	✓	NR	NR	✓	NR	NR	✓	Fair
Arzamani, 2012 [29]	✓	NR	✓	✓	✓	✓	NR	✓	✓	NA	✓	Good
Salehi, 2024 [30]	✓	✓	✓	✓	✓	✓	✓	✓	✓	✓	✓	Good
Torabi, 2009 [31]	✓	NR	✓	✓	✓	NR	NR	✓	NR	NR	✓	Fair
Rassi, 2012 [32]	✓	✓	✓	✓	✓	✓	✓	✓	✓	✓	✓	Good
Mohebali, 2016 [34]	✓	✓	✓	✓	✓	✓	✓	✓	✓	✓	✓	Good
Taslimian, 2019 [35]	✓	NR	✓	✓	✓	NR	NR	✓	NR	✓	✓	Fair
Chinikar, 2012 [36]	✓	✓	✓	✓	✓	✓	✓	✓	✓	✓	✓	Good
Saghafipour, 2019 [37]	✓	NR	✓	✓	✓	✓	✓	✓	✓	✓	✓	Good
Janati Pirouz, 2015 [39]	✓	NR	✓	✓	✓	NR	✓	✓	NR	NR	✓	Fair
Rashidi, 2013 [40]	✓	NR	✓	✓	✓	NR	✓	✓	NR	✓	✓	Good
Abedi, 2014 [41]	✓	✓	✓	✓	✓	✓	✓	✓	NR	NR	✓	Good
Heidari, 2015 [42]	✓	NR	✓	✓	✓	NR	NR	✓	NR	✓	✓	Fair
Arzamani, 2016 [43]	✓	NR	✓	✓	✓	✓	NR	✓	✓	✓	✓	Good
Reaghi, 2016 [44]	✓	NR	✓	✓	✓	✓	✓	✓	NR	✓	✓	Good
Arzamani, 2017 [45]	✓	✓	✓	✓	✓	✓	✓	✓	✓	✓	✓	Good
Firoozfar, 2021 [46]	✓	✓	✓	✓	✓	NA	NA	✓	NR	✓	✓	Good
Firoozfar, 2019 [47]	✓	✓	✓	✓	✓	NA	NA	✓	NR	✓	✓	Good
Rafinejad, 2000 [49]	✓	NR	✓	✓	✓	NA	NA	✓	NR	NR	✓	Fair
Rajabzadeh, 2015 [50]	✓	NR	✓	✓	✓	NR	✓	✓	NR	NR	✓	Fair
Firoozfar, 2019 [51]	✓	NR	✓	✓	✓	NA	NA	✓	✓	✓	✓	Good
Moshaverinia, 2016 [52]	✓	NR	✓	✓	✓	✓	✓	✓	NR	✓	✓	Good

*Note:* Q1: Is the target population of the study clearly defined and described? Q2: Are competing alternatives clearly described? Q3: Is the research question clearly formulated and well‐structured? Q4: Does the chosen study design align appropriately with the stated research objective? Q5: Is the selected time frame suitable for the context and aims of the study? Q6: Is the analytical perspective adopted in the study relevant and justified? Q7: Are all important variables, whose values are uncertain, appropriately subjected to sensitivity analysis? Q8: Do the conclusions of the study logically follow from the reported data and findings? Q9: Does the article address the potential generalizability of the results to other populations or settings? Q10: Is there a clear disclosure regarding the absence of conflicts of interest between researchers and funders? Q11: Are ethical considerations and equity‐related issues thoughtfully discussed? Good: Meets 8–11 criteria, Fair: Meets 4–7 criteria, Poor: Meets 0–3 criteria. Some criteria (e.g., sensitivity analysis) are less relevant for purely descriptive epidemiological studies; these were scored as NA or NR where appropriate.

Abbreviations: NA = not applicable, NR = not reported.

^∗^Ref. No: reference number.

Exclusion criteria were as follows:

• Duplicate publications.
• Articles lacking full‐text availability.
• Studies unrelated to the research objectives or not relevant.
• Non–peer‐reviewed sources, including editorials, commentaries, and conference abstracts.

The initial screening was performed based on titles and abstracts, followed by a detailed full‐text review using the criteria above. Disagreements were resolved through discussion. Furthermore, studies were appraised for quality based on geographic relevance, study design, sample size, and methodological rigor to ensure the inclusion of valid and reliable evidence. As the present study is a narrative review, no statistical software was used for meta‐analysis, and descriptive synthesis was employed to summarize findings.

## 3. Results

### 3.1. Sand Fly‐Borne Diseases

#### 3.1.1. Cutaneous Leishmaniasis (CL)

North Khorasan Province is a primary endemic area for CL in Iran. Human CL cases have been documented across all districts of the province, with occurrences throughout the year, predominantly with peaks in autumn (September–November) when sand fly activity is highest due to favorable temperatures and humidity [21–24].

From 2006 to 2022, the cumulative incidence of CL in North Khorasan Province was 426 cases per 100,000 people (3680 cases in a population of 863,000). Although CL was recorded in all counties of this province, the spatial analysis shows that Esfarayen county had the highest incidence, with 18.50 cases per 1000 people (1325 cases in a population of 71,612). While Maneh–Samalqan county had the lowest incidence at 0.074 cases per 1000 people (56 cases in a population of 75,645), it should be mentioned that most patients reside in small rural communities or have a history of travel to endemic regions of Iran [21–25]. In the meantime, some new cases of atypical disseminated CL in opium abusers have been reported in Maneh–Samalqan and Esfarayen counties in the province [26, 27].

In previous studies, two species of *Leishmania*, including *Leishmania major* as the causative agent of zoonotic cutaneous leishmaniasis (ZCL) and *Leishmania tropica*, the causative agent of anthroponotic cutaneous leishmaniasis (ACL), were identified from human lesions in the province. According to molecular and epidemiological research on CL in North Khorasan, *Rhombomys opimus* and *Phlebotomus papatasi* (Diptera: Psychodidaea) were reported as the main reservoir and vector for ZCL in the province [24, 28] (Table 2).

**TABLE 2:** Different studies conducted on arthropod‐borne diseases in North Khorasan Province, Iran.

Arthropod‐borne disease/health threat	Key findings	Sample size/data source	Detection method	Reference
CL	The disease in Esfarayen is of zoonotic type, with *R. opimus* as the reservoir and *P. papatasi* as vector	NA	Epidemiological investigation	[28]
	All 4 isolates from lesions of patients were *L. major*	4 humans	PCR–RFLP	[21]
	The 2831 patients with CL were reported during 2006–2013	2831 humans	Microscopic examination	[22]
	*L. tropica* and *L. major* were confirmed as agents of CL in Esfarayen/Jajarm	96 human samples	PCR	[23]
	*L. major* was identified as the agent of CL	849 human samples	Microscopic examination	[25]
	The isolated *L. major* strains were similar to other Iranian isolates in North Khorasan	200 human samples	PCR	[24]

VL	The 24 infants, children, and adults with VL reported in the North Khorasan Province during 2010 to 2018	24 human cases	Epidemiological investigation	[30]
	The 47 cases (2.92%)were positive for *L. infantum*	1608 human blood samples	Direct agglutination test (DAT)	[31]
	All four dogs were positive *L. infantum*	4 dogs’ blood samples	Direct agglutination test (DAT)	[31]
	The report of CL in a young woman infected with *L. infantum*	One human case	Direct agglutination test (DAT)	[54]
	The 164 cases were positive *L. infantum* among 1990–2010	164 human cases	Direct agglutination test (DAT)	[29]
	Of 59 female *P. kandelakii*, 2 (3.4%) were naturally infected with *L. infantum*	2088 sand flies	Nested PCR	[32]
	The fox and jackal showed anti‐*L. infantum* antibodies with titers of 1:320–1:20480 in DAT	84 wild canines	DAT	[34]

DCL	The remarkable occurrence of VL (29.7%) in asymptomatic sheepdogs	37 dogs	PCR	[35]
	An atypical case of DCL in a 26‐year‐old HIV‐positive man	On human sample	PCR–RFLP	[27]
	The report of atypical DCL in an opium abuser	On human sample	Microscopy; PCR	[26]

Theileriosis	*Theileria spp*. identified in 74 (82.2%) of blood samples	90 blood samples of sheep	PCR	[40]
	*H. marginatum turanicum* and *R. turanicus* were infected with *Theileria spp*.	434 ticks	PCR	[40]

CCHF	No CCHF infection was seen in North Khorasan	16 human samples	ELISA; RT‐PCR	[36]
	The CCHFV infection was reported in *H. anatolicum* and *Rh. sanguineus*	62 ticks	ELISA; RT‐PCR	[37]
	The CCHFV‐specific IgG antibodies were confirmed in sheep and goats	136 sheep and goats		[37]

Q fever	Seroprevalence of *C. burnetii* at the animal level was 28.7% for camel populations	167 camels	ELISA	[39]

Tick‐borne Encephalitis	No TBE infection reported in birds	10 birds	PRNT	[9]

Equine Piroplasmosis	*H. a. excavatum* and *R. bursa* had a positive reaction for *T. equi*	Blood smears of 100 horses and their Ixodidae ticks	Microscopy; PCR	[41]

West Nile fever	The WNV was identified in 20% of birds	10 birds	PRNT	[9]

Chikungunya	The CHIKV was confirmed in mosquitoes	371 mosquito female samples	RT‐PCR	[8]
	The CHIKV was confirmed in mosquitoes	371 mosquito female samples	RT‐PCR	[8]

Dirofilariasis	*D. immitis* confirmed from jackals (*Canis aureus*) in North Khorasan	45 Jackals	PCR	[42]

*D. caninum* infection	*D. caninum* confirmed in dogs	32 stray dogs	Morphological identification	[43]

Fascioliasis	*F. hepatica* was found in cattle from this region of Iran	30 cattle	PCR	[44]

Pediculosis	*The P. capitis* infestation was determined to be 7.7%	1475 students	Visual inspection	[50]
	A total of 28 cases (3.2%) were found to be infected with head lice	867 humans	Microscopic confirmation	[51]

Scorpionism	During 2007–2018, 540 scorpionism cases were admitted to hospitals	All residents in the province	Hospital and health centers documents	[46]
	Five species of scorpions identified in this province	143 scorpions	Morphological identification	[47]

Spiders	Four species of *Latrodectus* spp. of spiders recorded from this province	216 live specimens of spiders	Morphological identification	[49]

Myiasis	The third‐stage larvae of *Gasterophilus pecorum* were found in horses in this region	80 Turkmen horses	Morphological identification	[52]

Hymenolepiasis	The hymenolepiasis in rodents has been reported in this province	37 rodents	Necropsy; helminth morphological analysis	[45]

*Note: C. = Coxiella*, CHIKV = Chikungunya virus, *D. = *Dipylidium, *F. = fasciola, P. = Pediculus*, *H.* = Hyalomma, *P. = Phlebotomus*, *L.* = *Leishmania*, *tropica, R. = Rhombomys, Rh.* = *Rhipicephalus*.

Abbreviations: CCHF = Crimean–Congo hemorrhagic fever, CL = cutaneous leishmaniasis, DCL = disseminated cutaneous leishmaniasis, NA* = *not applicable, TBE = tick‐borne encephalitis, VL = visceral leishmaniasis, WNV = West Nile virus.

#### 3.1.2. Visceral Leishmaniasis (VL)

Several studies have shown that VL is endemic in some areas of North Khorasan Province. Some counties such as Shirvan, Maneh–Samalqan, Bojnurd, and Raz Jargalan were defined as endemic foci in the province. Arzamani et al. retrospectively evaluated 164 infants, children, and adults with VL based on all hospitals in the province and all documents in the health centers of the province, with prevalence ranging from 1.00 to 2.50 per 100,000 population in North Khorasan Province during 1990–2010. The disease has been reported from different counties of the province [29]. Furthermore, Salehi et al. also reported 24 diagnosed patients with VL, with prevalence ranging from 0.0 to 1.1 per 100,000, between 2010 and 2018 [30]. Along the same lines, Torabi et al. reported that thirty‐eight (2.36%) out of 1608 collected blood samples from Bojnurd county, the capital of North Khorasan Province, had anti‐Leishmania antibodies at titer 1:800 and that nine (0.56%) were just positive at 1:3200 by DAT [31]. According to previous studies, the majority of VL cases are reported in late spring and summer, aligning with the breeding cycles of *Phlebotomus* spp. sand flies [32, 33]. Likewise, Rassi et al. reported the first detection of *Leishmania infantum* in *Phlebotomus kandelakii* using molecular methods in North Khorasan. They found that, out of 59 female *P. kandelakii*, 2(3.4%) were naturally infected with *L. infantum* [32].

In the reservoir host (jackals and dogs), wild canine leishmaniasis was shown to be a sylvatic transmission cycle of VL in different counties of the province. Amastigotes of *L. infantum* have been observed in foxes, jackals, and necropsied domestic dogs [31, 34, 35] (Table 2).

### 3.2. Tick‐Borne Diseases

#### 3.2.1. Crimean Congo Hemorrhagic Fever

In a cross‐sectional study conducted by Chinikar et al. among slaughterhouse workers in northeast Iran during 2004 and 2005, IgG against CCHF was not found in North Khorasan [36].

In a study conducted by Saghafipour et al. in 2019, the CCHFV infection rate was 8.1% (*n* = 5 ticks infected out of 62) in ticks collected in North Khorasan Province. The infected species of ticks were *Hyalomma anatolicum* (*n* = 3) and *Rhipicephalus sanguineus* (*n* = 2). Additionally, the study revealed that the seasonal pattern of tick activity in this province is in spring and early summer [37].

Furthermore, serological tests conducted on domestic animals were positive in 15.4%. Serologic tests detected specific IgG antibodies of this virus in 16.2% and 19.2% of sheep and goats, respectively, while all sera collected from cows were negative. Moreover, serologically positive livestock in all counties and ticks infected with the CCHFV genome were identified in Bojnurd and Maneh–Samalqan counties [37]. Along similar lines, a serosurvey conducted in Khorasan Province, northeastern Iran (2003–2005), revealed CCHFV antibodies in 77.5% of sheep (*n* = 298) and 46% of goats (*n* = 150) among 448 livestock samples tested [38] (Table 2).

#### 3.2.2. Q Fever

There was no report of *Coxiella burnetii*, the causative agent of Q fever in humans residing in North Khorasan Province. In addition, the seroprevalence of *C. burnetii* antibodies in animals (camels) of North Khorasan Province was detected to be 25.4% [39] (Table 2).

#### 3.2.3. Theileriosis and Piroplasmosis

There were some studies on theileriosis and piroplasmosis in animals such as sheep and camels in North Khorasan. The *Theileria* spp. infection was reported in 82.2% of collected sheep sera using seminested PCR in North Khorasan Province in 2013. *Theileria ovis*, *Theileria lestoquardi*, and mixed infection were detected in 70%, 5.5%, and 6.6% of samples, respectively. Furthermore, three pools of *H. marginatum turanicum* salivary glands were infected with *T. ovis* and *T. lestoquardi*, and one pool of *R. turanicus* was infected with *T. ovis*. It can be suggested that the prevalence of *T. ovis* is higher than *T. lestoquardi* and that *H. marginatum turanicum* and *R. turanicus* are possible vectors of *T. lestoquardi* and *T. ovis* in North Khorasan Province [40]. Additionally, equine piroplasmosis with *Theileria equi* and *Babesia caballi* has been reported in horses and ticks, including *Hy. marginatum marginatum*, *Hy. anatolicum excavatum*, *Rh. bursa*, *Hy. marginatum turanicum*, and *Hy. anatolicum anatolicum* in this province [41] (Table 2).

### 3.3. Mosquito‐Borne Diseases

#### 3.3.1. West Nile Fever

There was only one report about West Nile virus (WNV) in North Khorasan. Bakhshi et al. have reported evidence of WNV infection among birds in North Khorasan Province [9]. The results obtained using micro virus neutralization tests have shown that a golden eagle collected in North Khorasan Province was positive for WNV infection (Table 2).

#### 3.3.2. Chikungunya

There is only one report about chikungunya virus (CHIKV) in 2020, which shows that the virus is circulating in some areas of North Khorasan Province. In a viral detection method using the BioMark microfluidic system by Bakhshi et al. in 2020, the presence of CHIKV was confirmed in five pools of mosquitoes collected in North Khorasan Province [8]. The results indicated that *Culiseta longiareolata*, *Culex tritaeniorhynchus*, and *Anopheles maculipennis* were infected with this virus in Shirvan and Raz Jargalan, located in North Khorasan Province (Table 2).

#### 3.3.3. Dirofilariasis

There was only one report of this disease in North Khorasan Province in the animal population.

In a study conducted between 2013 and 2014, the carcasses of 45 golden jackals (Canis aureus), 16 foxes (*Vulpes vulpes*), 15 dogs (*Canis familiaris*), and one wolf (*Canis lupus*) were necropsied. By gross examination, adult filarial nematodes were found in the cardiovascular system of four jackals (8.9%). The morphological characteristics of the recovered heartworms were compatible with *Dirofilaria immitis* [42] (Table 2).

### 3.4. Parasites

#### 3.4.1. *Dipylidium caninum*, Fascioliasis, and Hymenolepiasis

In an investigation conducted in 2016, the prevalence and intensity of zoonotic parasites among stray dogs from Bojnurd county, the capital city of North Khorasan Province, were evaluated. This study showed the presence of at least one helminth in 87.5% of stray dogs (*n* = 28 dogs infected out of 32). In addition, 37.5% of examined dogs (*n* = 12 dogs infected out of 32) were infected with *Dipylidium caninum* [43]. Furthermore, evidence of the prevalence of fascioliasis in cattle [44] and hymenolepiasis in rodents has been reported in this province [45] (Table 2).

### 3.5. Other Diseases and Complications Caused by Arthropods

#### 3.5.1. Scorpion Stings (Scorpionism)

Recent scorpion stings in North Khorasan Province were studied over 12 years. Out of 540 cases admitted to various health centers in Bojnurd, Shirvan, Esfarayen, Jajarm, and Raz Jargalan counties, 44 individuals required hospitalization. The majority of incidents (58.4%) took place indoors [46] (Table 2).

A faunistic study conducted in North Khorasan showed that all of the collected scorpions belonged only to the Buthidae family. They were also classified into four genera (*Androctonus*, *Mesobuthus*, *Odontobuthus*, *Orthochirus*) and five species: *M. eupeus* (59.44%), *A. crassicauda* (16.78%), *O. doriae* (12.59), *M.* (*Olivierus*) *caucasicus* (9.09%), and *O. farzanpayi* (2.10%) [47].

#### 3.5.2. Spider Bites

In a study carried out by Rafinejad et al., 195 cases of spider bites were reported in North Khorasan Province spanning a period of 4 years (1994–1997). The identified species were *Latrodectus tredecimguttatus*, *L. dahli*, and *L. geometricus*, with *L. tredecimguttatus* reported as the most prevalent spider species in this province [48, 49].

#### 3.5.3. Head Lice Infestation (Pediculosis)

Pediculosis is prevalent in people of all counties of this province. For instance, pediculosis was reported among elementary school students of Bojnurd City. The infestation rate was 7.7%, with females experiencing a higher infestation rate than males (12.4% versus 2.9%, respectively). This study showed a significant relationship between pediculosis and sex, family size, and public health facilities [50]. In 2019, a cross‐sectional study among Kormanj tribes in the province found a 3.2% infestation rate of head lice. Significant statistical association was noted between head lice infestation and sex, while no significant difference was found between infestation and the public health facilities [51] (Table 2).

#### 3.5.4. Myiasis

In an investigation related to myiasis, the cause of death of nine mares of 80 Turkmen horses was determined in Maneh and Samalghan County, located in North Khorasan Province. Parasitological tests revealed the presence of larvae of *Gasterophilus pecorum* in their third stage. This study was the first report of infection of horses with these botflies in the country [52] (Table 2).

## 4. Discussion

This review goes beyond a descriptive compilation of previous reports and provides an analytical synthesis of the epidemiological patterns, seasonal dynamics, and public health significance of ARHTs in North Khorasan Province. The findings highlight a complex interplay between the region’s semiarid ecology, livestock dynamics, and vector‐borne pathogen transmission. Overall, the evidence suggests that North Khorasan is not merely a repository of isolated cases but rather a dynamic transition zone where multiple transmission cycles coexist and potentially interact.

CL remains the most significant public health burden in North Khorasan, with incidence rates notably exceeding the national average [22, 49, 53]. The dominance of *L. major* and *L. tropica*, alongside the well‐documented reservoir role of *Rh. opimus* and vector *Ph. papatasi*, indicates a stable zoonotic and anthroponotic transmission cycle [24–28]. The seasonal peak in autumn aligns with both sand fly activity and agricultural harvest periods, suggesting that occupational exposure during farming is a primary driver of human infection [22, 25]. Furthermore, the emergence of atypical disseminated CL in opium users and HIV‐positive individuals in counties like Esfarayen and Maneh–Samalqan signals a potential shift toward immunocompromised susceptibility, necessitating targeted screening in high‐risk subpopulations [26, 27].

VL, although less frequently reported in humans, poses a latent threat in North Khorasan [29, 54]. The detection of *L. infantum* in wild canines (foxes, jackals) and domestic dogs, along with naturally infected *Ph. kandelakii*, confirms the presence of a sylvatic transmission cycle in this province [27, 31–35]. The relatively low human seroprevalence (1.00–2.50 per 100,000) [29, 30] may underestimate the true burden due to asymptomatic infections or diagnostic limitations, a common issue in VL surveillance. The presence of infected wild reservoirs suggests that VL is entrenched in the ecosystem, and any change in land use or human–wildlife contact could trigger outbreaks. In North Khorasan Province, like other endemic areas of VL in Iran such as Ardabil, East Azerbaijan, and Fars Provinces [55], the conditions are available for the completion of the chain of infection. According to reports, various species of vectors and reservoirs of the disease are present in this province. Therefore, if human hosts do not take preventive measures such as personal protection, they are exposed to the bites of sandflies infected with the *Leishmania* parasite and become infected.

CCHF is a tick‐borne zoonosis caused by the Nairovirus genus. The disease exhibits high case‐fatality rates (up to 50%), particularly in hospital settings. Iran has remained an endemic focus since the first reported human case in 1970, with an average of 65 confirmed annual cases reported nationally over the past two decades [56]. While CCHF occurs in 26 provinces, six provinces, including Sistan and Baluchestan, Isfahan, Fars, Tehran, Golestan, and Khuzestan, account for the majority of cases [57]. The province exhibits a striking disparity between high seroprevalence in livestock and low reported human cases for diseases like CCHF and Q fever [58–61]. For CCHF, the infection rate of 8.1% in ticks (*Hyalomma anatolicum* and *Rhipicephalus sanguineus*) and hyperendemic circulation in sheep (77.5%) and goats (46%) [37, 38] indicate intense viral circulation. The absence of reported human cases and negative serology in slaughterhouse workers [36] contrasts sharply with neighboring provinces. This discrepancy likely reflects a “surveillance gap” rather than true absence of risk. Occupational exposure among slaughterhouse workers should be higher than in the general population; thus, negative results might indicate effective protective practices in specific settings or, more likely, underreporting of mild cases. Similarly, the high seroprevalence of *Coxiella burnetii* in camels (25.4%) [39], despite no reported human Q fever cases, underscores the need for enhanced diagnostic surveillance in febrile patients with occupational animal contact. The high animal prevalence acts as a warning signal for potential zoonotic spillover, especially given the province’s role in livestock transit from neighboring regions.

Although no human cases of theileriosis are reported in North Khorasan Province, there are some studies on theileriosis in animals such as sheep and camels in the province. The *Theileria* spp. infection was reported in 82.2% of collected sheep sera using seminested PCR in North Khorasan Province in 2013 [41]. In a review conducted by Soosaraei et al., the prevalence of theileriosis among domestic herbivores was reported as 19%. Furthermore, they reported that the average maximum prevalence occurred in Razavi Khorasan (60.4%), located in the south of North Khorasan Province [62].

The detection of CHIKV in mosquito pools (*Culiseta longiareolata*, *Culex tritaeniorhynchus*, and *Anopheles maculipennis*) [8] and WNV in birds [9] highlights the province’s susceptibility to arboviral incursions. Although human cases have not been widely reported, the presence of competent vectors and infected animal hosts creates an epidemic potential. The seasonal transmission window for CHIKV (April–October) [8] overlaps with peak agricultural activities, increasing human‐vector contact. Climate change projections for North Khorasan, including rising temperatures and erratic rainfall [18–20], may extend the activity periods of these vectors, potentially shifting the transmission seasons earlier or later than historically observed. This ecological shift necessitates adaptive surveillance strategies that monitor vector population dynamics in real time.

Beyond infectious diseases, nuisance arthropods such as scorpions and spiders contribute significantly to morbidity. The predominance of *Mesobuthus eupeus* and indoor stings [46, 47] reflects adaptation to human dwellings, likely exacerbated by urbanization and housing conditions. Pediculosis (head lice) prevalence in students and tribal populations [50, 51] correlates with socioeconomic factors, overcrowding, and limited access to public health facilities, indicating that these infestations are markers of social inequality rather than just biological phenomena.

It is worth mentioning that it is crucial to interpret these findings with caution due to inherent limitations in the underlying data. First, the heterogeneity in study designs, diagnostic methods (e.g., microscopy vs. PCR), and sampling frames introduces potential bias. For instance, seroprevalence estimates for CCHF and Q fever vary widely depending on the population sampled (slaughterhouse workers vs. general livestock), making direct comparisons difficult. Second, the retrospective nature of many included studies limits the ability to establish temporal trends accurately. Third, the absence of human cases for certain zoonoses (e.g., Q fever, human CCHF) may result from underdiagnosis or lack of active surveillance rather than true absence. Future studies should prioritize standardized, active surveillance protocols and molecular characterization of pathogens to clarify transmission dynamics.

## 5. Conclusion

The available evidence indicates that North Khorasan Province provides ecological and occupational conditions that allow several ARHTs to persist. Among these threats, CL appears to play the most prominent role in the province. Its transmission cycle is relatively well understood and involves established vector and reservoir populations, while seasonal increases in cases coincide with periods of greater outdoor activity. VL also represents an ongoing but less documented threat, with transmission maintained through wild canines and domestic dogs; however, the lack of comprehensive canine surveillance limits accurate assessment of the true human disease burden. The detection of Crimean–Congo hemorrhagic fever virus in ticks and livestock further indicates that the ecological conditions necessary for human infection already exist, even though confirmed human cases have not been documented in the province.

Overall, these observations highlight the need for sustained surveillance and closer collaboration between human and veterinary health sectors to support early detection and improve the control of arthropod‐borne diseases in North Khorasan Province.

## Author Contributions

Abedin Saghafipour, Hasan Bakhshi, and Kourosh Arzamani conceptualized and wrote the study design and proposal. Hasan Bakhshi, Abedin Saghafipour, Hamidreza Shoraka, and Fatemeh Kouhestani carried out the data collection and methodology. Abedin Saghafipour, Kourosh Arzamani, Aboozar Soltani, and Jalil Nejati performed data collation and analyses. Abedin Saghafipour and Alireza Omidi Oskouei performed final writing–review and editing. All authors contributed to the final draft writing and review.

## Funding

The study did not receive any funding from external sources.

## Ethics Statement

The study protocol was reviewed and approved by the Ethical Review Committee of Qom University of Medical Sciences (IR.MUQ.REC.1397.017).

## Conflicts of Interest

The authors declare no conflicts of interest.

## Data Availability

The data that support the findings of this study are available from the corresponding author upon reasonable request.
